# Lung Cancer: A Classic Example of Tumor Escape and Progression While Providing Opportunities for Immunological Intervention

**DOI:** 10.1155/2012/160724

**Published:** 2012-07-29

**Authors:** Martin R. Jadus, Josephine Natividad, Anthony Mai, Yi Ouyang, Nils Lambrecht, Sandor Szabo, Lisheng Ge, Neil Hoa, Maria G. Dacosta-Iyer

**Affiliations:** ^1^Research Service, VA Long Beach Healthcare System, 5901 E. 7th Street, Long Beach, CA 90822, USA; ^2^Department of Pathology and Laboratory Medicine, University of California, Irvine, Irvine, CA 92697, USA; ^3^Chao Family Comprehensive Cancer Center, UC Irvine School of Medicine, University of California, Irvine, Orange, CA 92868, USA; ^4^Health Care Group, Department of Diagnostic and Molecular Medicine, Pathology and Laboratory Medicine Service, VA Long Beach Healthcare System, 5901 East 7th Street, Box 113, Long Beach, CA 90822, USA

## Abstract

Lung cancers remain one of the most common and deadly cancers in the world today (12.5% of newly diagnosed cancers) despite current advances in chemo- and radiation therapies. Often, by the time these tumors are diagnosed, they have already metastasized. These tumors demonstrate the classic hallmarks of cancer in that they have advanced defensive strategies allowing them to escape various standard oncological treatments. Immunotherapy is making inroads towards effectively treating other fatal cancers, such as melanoma, glioblastoma multiforme, and castrate-resistant prostate cancers. This paper will cover the escape mechanisms of bronchogenic lung cancer that must be overcome before they can be successfully treated. We also review the history of immunotherapy directed towards lung cancers.

## 1. Introduction

Approximately 12.5% of the newly diagnosed cancers in the world are lung cancers (World Cancer Research Foundation International). Lung cancer leads the world in newly diagnosed cancers: 1.6 million new cases were diagnosed in 2008. In the USA, about 225,000 newly diagnosed patients are annually reported. Bronchogenic lung cancers (LCs) have very fast growth rates. This basic aspect of lung cancer biology makes them sensitive to chemo- and radiation-based therapies for a temporary palliative treatment. These treated lung tumors will eventually relapse because a number of cancer clones or “cancer initiating cells” have escaped the initial therapy. These cells are selected and will return with enhanced resistance to therapeutic modalities. Additional adjuvant treatments are needed to eliminate those remaining cells that survived the initial therapy. Immunotherapy provides the opportunity to destroy the residual lung cancer cells that chemotherapy and radiation miss and may attack the so-called “cancer stem cells.” By activating the host immune lymphocytes, these cells can theoretically infiltrate into those remaining pockets of tumor cells and eliminate them. Progress has been made using immunotherapy to successfully improve the survival of some patients with other fatal types of cancer, such as glioblastoma multiforme and castrate-resistant prostate cancer [1, 2]. Some of the lessons learned from those cancers can be directly applied to cure lung cancer, too.

## 2. Derivations of Lung Cancer

Lung cancers (LCs) are very aggressive tumors derived from different cell types. The incidence of LC in the western world rose precipitously during the 20th century due to increased prevalence of smoking. The prevalence of new lung cancer is dropping in those western countries that successfully discourage smoking; however, the incidence of LC is now rising in those developing countries that see smoking as an easy form of tax revenues. These smoking-induced lung cancers are predominantly caused by Ras mutations. Tobacco smoke contains many carcinogens [3] including very complex aromatic hydrocarbons (benzopyrenes) and other organic compounds such as nitrosamines. The classic example of cancer induction due to chronic carcinogenic exposure usually involves tobacco-derived carcinogens. Once these carcinogens enter healthy cells they induce genetic mutations, which lead to oncogenic transformation. Besides chemical carcinogens, asbestos, radioactive radon, polonium, and plutonium can also contribute to the formation of LC. Finally, there are individuals who can spontaneously develop lung cancer without any known carcinogenic exposures. Asian women, usually of Japanese descent, possess epidermal growth factor receptor (EGFR) mutations [4, 5]. Some younger men have an echinoderm microtubule-associated protein-like 4 (EML4) and anaplastic lymphoma kinase (ALK) translocation (EML4-ALK) [6, 7], which causes their cancer.

Small-cell lung cancer (SCLC) arises from neuro-endocrine cells, the “Kulchitsky cells” of the lungs. These cancers represent about 20–30% of all lung cancer diagnoses in the USA. This type of lung cancer was previously called “oat cell” cancer. These cells make polypeptide hormones and are characterized by dense core neurosecretory granules. These small-cell lung cancers are different from the non-small-cell lung cancers (NSCLCs). NSCLC includes the adenocarcinomas, squamous and large cell cancers usually arise from alveolar cells. Adenocarcinomas come from basal bronchial cells and type II pneumocytes that arise in the periphery of the lung, while the squamous type lung cancers arise from the bronchial epithelial cells located more centrally. The incidence of squamous lung cancer is dropping in the USA and it has now been overtaken by adenocarcinomas, perhaps due to the reformulation of cigarettes back in the 1970s to contain filters. These filters prevent the larger particulate matter containing the carcinogens from getting into the lungs. The smaller carcinogenic smoke particles still reach into the depths of the lungs. Thus, the percentage of non-small-cell lung cancers (NSCLCs) is now trending towards a more adenocarcinomous type of cancer. The two types of spontaneous lung cancers due to nonsmoking causes are predominantly adenocarcinomas. These types of cancers are thought to be a totally different type of lung cancer when compared to those adenocarcinomas generated by smoking [8]. 

## 3. Pathological Characteristics of Lung Cancers

**Figure 1:**
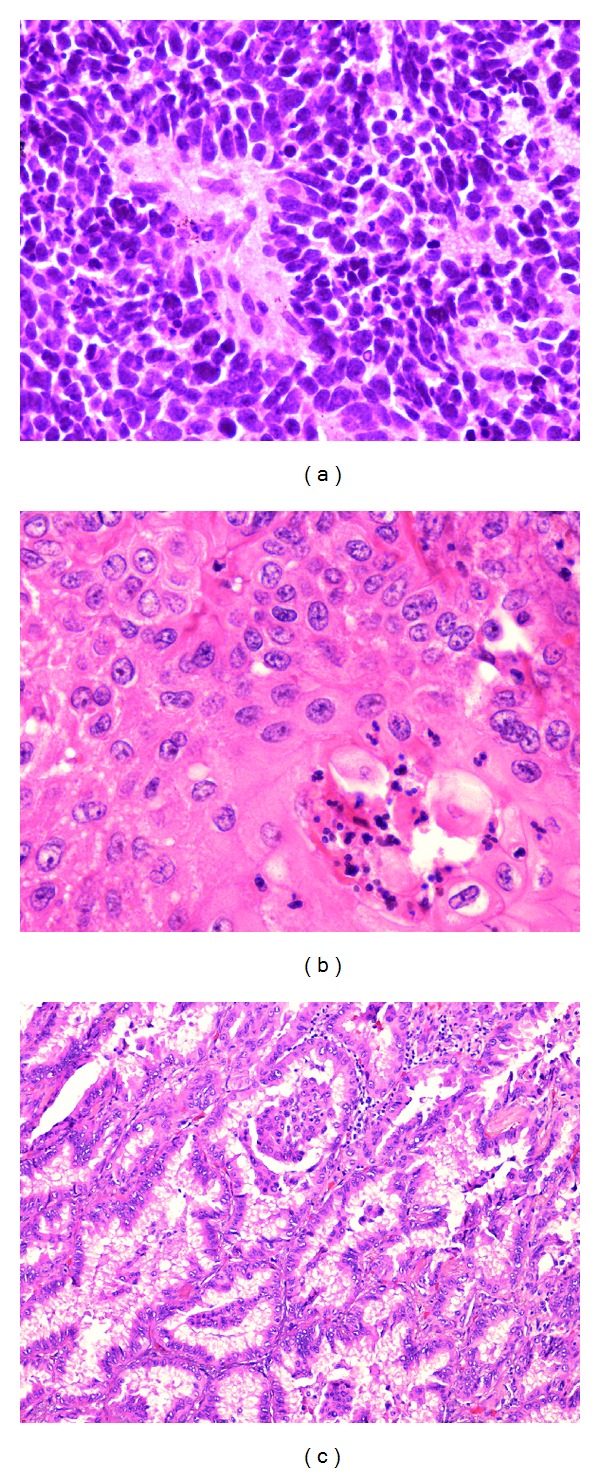
Representative histological micrographs of the most common lung cancers. (a) Small-cell lung cancer taken with a 40x objective lens. (b) Squamous cell lung cancer visualized with a 40x objective lens. (c) Adenocarcinoma lung cancer seen using a 10x objective lens.

Lung cancers are mostly bronchogenic carcinomas. Small-cell carcinoma consists of round, oval, and spindle-shaped malignant small cells with scant cytoplasm, ill-defined cell borders, and finely granular nuclear chromatin. Nuclear molding is prominent. Mitotic count is high and usually presents with extensive necrosis (Figure 1(a)). Squamous cell carcinoma is characterized by infiltrating nests of malignant epithelial cells with keratinization and/or intercellular bridges (Figure 1(b)). Adenocarcinoma typically shows glandular differentiation with acinar, papillary, bronchioalveolar, solid, or mixed pattern with mucin production (Figure 1(c)). Ruffini and coworkers [9] presented data which showed that *in situ* adenocarcinomas were infiltrated by lymphocytes about 25% of the time. In contrast, only 5% of SCLCs had lymphocytic infiltrates. SCLCs have a higher proliferative rate than the adenocarcinomas. 

## 4. Survival of Lung Cancer Patients

Because lung cancer cells divide so rapidly, they are temporarily treatable by standard oncological therapies. Localized radiation is applied if the cancer is still physically found within the affected regions of the lung. Adenocarcinomas and squamous cell carcinomas are usually surgically removed as a first step. By the time small-cell lung cancers are discovered they have usually disseminated systemically and so tend to have a poorer prognosis when compared to early detection of NSCLC. Systemic chemotherapy after surgical resection is given. Localized LCs have the best prognoses, whereas metastatic LCs have the worst survival. Lung cancer patients have an overall 5-year survival rate of 16% (2011 American Cancer Society). The survival statistics indicate that these tumor cells have a variety of escape mechanisms that counteract current therapeutic interventions. These escape pathways include antiapoptosis, drug resistance, and immunodefensive routes.  Table 1 shows the various escape Pathways that lung cancers can use and will be discussed in detail.

## 5. Escape Mechanisms

### 5.1. Antiapoptosis Genes

Radiation and most chemotherapeutic drugs kill tumor cells via apoptosis. Many “cancer stem-like” cells are radio- and chemotherapeutic resistant [10–13]. Lagadec and coworkers [14] showed with breast cancer stem cells that upon radiation treatment reprograms those remaining cells. So when these cancers relapse they will come back with enhanced antiapoptotic gene expression. These altered profiles include increased bcl2, survivin, and livin, which makes these relapsing cancer cells more resistant to drugs that were previously used to treat the initial LC [15–22]. Bcl2 is highly expressed in SCLCs and somewhat less expressed in squamous lung cancers (about one third as much as found in SCLCs), [23]. Survivin is highly expressed in most NSCLCs [21]. These mutations make the cancer cells more resistant to therapy when treatments are given. Bcl2 and survivin are both induced by PGE_2_ [24, 25] (see Section 5.3.1(a)). Many of these genetic alterations and mutations within LC have been directly attributed to the actions of carcinogens. Joseph et al. observed that SCLC lost production of caspase-1, -4, -8, and -10 (apoptosis effector molecules) [26]. It has been postulated that gene silencing via altered methylation profiles [27, 28] might be responsible for some of this loss of apoptosis executioner proteins within the SCLCs. 

Another type of mutation that frequently occurs within lung cancers would be point mutations within the p53 suppressor gene. P53 mutations within lung cancer lead to upregulation of Bcl2 while downregulating the pro-apoptotic expression of Bax [29]. Zöchbauer-Müller et al. [30] have reported that SCLCs and NSCLCs both contain these p53 mutations. There are other members of this family (e.g., p63, p73) [31, 32] that perform similar functions as p53. Mutations in these p53 family members prevent apoptosis execution functions, too. Consequently, these mutations prevent tumor cells from killing themselves in response to various therapies. 

Apoptosis is called the “silent death.” When cells die of classic apoptotic pathways, these dead apoptotic cells tend to be rapidly absorbed or “scavenged” by the adjacent cells. This prevents the local antigen-presenting cells (APCs) from having sufficient time to absorb this cellular debris in order to stimulate the immune system. In contrast, when cells die of a necrosis-dependent pathway, the immune system is now activated and begins an active immune response in response to the “danger signals” that have been released by the necrotic cells [33–37]. Danger signals include high gel mobility box-1 (HMGB1), uric acid, calreticulin, and the heat shock proteins (HSPs) [38]. Hence, drugs that kill tumor cells via this necrosis induction pathway provide better long-term effects by enhancing the immune system response to the cancer as they regress. There are cytotoxic drugs that can kill tumor cells via apoptosis and stimulate immune responses. A more appropriate terminology has been coined: “immunogenic” versus “nonimmunogenic” cell death to better represent the more nuanced version of this phenomenon [39, 40]. The judicious use of chemotherapeutic drugs that promote “immunogenic” apoptotic death may further improve chemotherapy against lung cancers by stimulating endogenous immune responses against the tumors.

### 5.2. Drug Resistance Genes

Another strategy that tumor cells use to evade the full effects of cytotoxic drugs is to export the chemotherapeutic drugs from the cells. Newly synthesized drug-resistant transmembrane proteins actively pump out the chemotherapeutic drugs that have entered into the cancer cell. These exporters effectively reduce the internal concentration of the drug, thereby preventing the full cytotoxic effects of the drug. The small amount of the drugs that remain may even activate cell repair mechanisms such as the cell stress pathways, which would allow heat shock proteins to remove and replace any damaged cellular components. This process may further promote drug resistance by the tumor cells. Lung cancers produce a variety of multidrug resistance proteins (MRPs) and P-glycoprotein [41–43]. In studies by Triller et al. [44] it was shown that when SCLCs relapse there are higher concentrations of MRP3 within the returning cancer cells.

CD133 has been reported to be a marker for various cancer stem cells, including NSCLC [12], although there is a report that their presumed NSCLC stem cells are aldehyde dehydrogenase-positive cells [45]. For brain cancers, CD133 is a marker of bioenergetic stress [46] and probably reflects the universal function of this molecule with many different cancer types. CD133 was initially described as a fluorescent dye reverse transporter [13, 47, 48]. Many fluorescent dyes have planar chemical structures that resemble many chemotherapeutic drugs. Therefore, if CD133+ cells are able to export chemotherapeutic drugs via this proposed mechanism, then these stem cells are naturally resistant to chemotherapy. Bertolini and colleagues [12] showed that CD133+ lung cancer cells resisted cytotoxic doses of cis-platinum. By asymmetric division, CD133+ “stem cells” differentiate into CD133-negative cancer cells. These CD133-negative cells are subsequently more likely to be killed by chemotherapy. Many early cancer treatments seem to be effective at first, with the bulk of the tumor disappearing. But over time the tumor returns. Thus, the new explanation is that “stem cells” are drug-resistant clones that manage to escape treatment and are responsible for reestablishing the tumor, after the therapy has stopped.

### 5.3. Immunoresistance Pathways

Lung cancers produce numerous defense strategies that allow them to hide from the immune system. These pathways include releasing soluble immunosuppressive agents, recruitment of suppressor-type cells, lack of immune stimulating molecules, and counterattack strategies. 

#### 5.3.1. Soluble Immunosuppressive Mediators

These mediators include prostaglandin (PGE) via cyclo-oxygenase, interleukin-10, other types of type-2 derived cytokines, transforming growth factor-*β*, and vascular endothelial growth factor, which inhibit *in situ* immune responses. These soluble mediators work in a variety of ways and most likely synergize with one other.

**(a) Prostaglandin**

Lung cancers produce several types of prostanoids and leukotrienes [49, 50], which are derived from arachidonic acid metabolism. Normal lung cells make little to no prostaglandin, whereas lung cancers (squamous, adenocarcinoma, small cell, and mixed lung cancers) produce elevated levels of prostaglandin E_2_ (PGE_2_). PGE_2_ is synthesized by either cyclo-oxygenase-1 (Cox-1) or -2 (Cox-2). Cox-1 is a constitutive enzyme that produces low levels of PGE_2_, while Cox-2 is the inducible form of the enzyme and produces much more PGE_2_. Many tumors [51], including lung cancers [52], overexpress the Cox-2 enzyme. Epidemiological studies have shown that a daily dose of aspirin helps reduce PGE_2_ production and lowers the incidence of a variety of cancers including lung cancers [53]. Prostaglandin E_2_ can bind to the 4 different prostaglandin receptors: EP1, EP2, EP3, and EP4 [54]. Lung cancer cell lines and *in situ* lung cancers express a variety of these receptors [55]. PGE_2_ regulates several aspects of lung cancer biology such as controlling angiogenesis, ERK stimulation, invasion, and proliferation [56–59]. PGE_2_ raises intracellular cyclic AMP levels [60, 61] within various immunocytes, which inhibits dendritic cells and prevents B, T, and NK cell effector functions.

**(b) Interleukin-10**

PGE_2_ also stimulates IL-10 production from a variety of cell types, macrophages, B cells, and T cells [62, 63]. Current dogma holds that interleukin 10 is a Th2 cytokine. IL-10 downregulates Th1 immunity (see below) and prevents effective antitumor immunity [64]. But there is a body of evidence which shows that IL-10 can actually assist in antitumor immune responses in a variety of tumor models, including lung cancer [65–67]. Thus, the role of IL-10 in tumor rejection is not as clear cut as previously thought and may be a double-edged sword. IL-10 may work in concert with other immunosuppressive agents or suppressor cells to produce protumor effects. LCs are known to produce Th2 polarization by releasing cytokines, IL-4, IL-5, IL-6, and IL-13, besides IL-10 [68–70]. These other cytokines along with PGE_2_ could either simply mask the effects of IL-10 or synergize with IL-10 to enhance the protumor activities. So a word of caution needs to be applied with IL-10.

**(c) Vascular Endothelial Cell Growth Factor**

All types of cancers, including lung cancers, make vascular endothelial cell growth factor (VEGF) [71–74]. Some LCs also express the VEGF receptors, suggesting that autocrine loops may control tumor cell growth [73, 75, 76]. VEGF promotes tumor angiogenesis by recruiting endothelial precursor cells from the blood to begin building new blood capillaries. These new blood vessels supply the tumor with oxygen and nutrients allowing the tumor to grow. Vascular permeability functions of endothelial cells are also enhanced by VEGF. VEGF also provides a mechanism by which the immune system is inhibited, by downregulating the functions of antigen-presenting cells (APCs) [77], adaptive immune responses are thereby prevented. The VEGF gene within the general population has several polymorphisms [78]. These polymorphisms may make certain individuals more susceptible to developing lung cancer and may explain why not all smokers develop lung cancer.

**(d) Transforming Growth Factor-*β***

Transforming growth factor-*β* (TGF-*β*) is another commonly overexpressed cytokine that performs multiple functions in tumor biology [79–81]. These activities include assisting tumor growth, improving angiogenesis, enhanced migration, fibrosis production, and increased proteolytic enzyme release, while simultaneously inhibiting the immune response. Lung cancers do overexpress several TGF-*β* isoforms-1, -2, and -3 while concurrently having mutated TGF-*β* receptors [82–88]. These mutations within the receptors prevent the negative signaling transduction pathways being delivered by TGF-*β* to the tumor cells. Simultaneously, this excess TGF-*β* influences the local microenvironment. TGF-*β* is a well known factor and is very good at inhibiting many aspects of cellular immunity (reviewed in [89, 90]). Like VEGF, there are reported polymorphisms in the TFG-*β*1 that make certain individual less susceptible to developing lung cancer [91].

#### 5.3.2. Immune Suppressor Cells

Many of these immunosuppressive agents listed above will recruit either Treg [91] or myeloid derived suppressor cells (MDSCs) [92]. The combined milieu of all of these immunosuppressive agents can bring about proper conditions that allow these suppressor cells to become the dominant immunologically active cells within lung cancers. The role of these types of suppressor cells is thought to be as a fail-safe mechanism by which the immune system is tightly regulated to prevent autoimmunity or other self-destruction. Lung cancer cells take full advantage of these suppressor cells.

**(a) T-Cell Biology**

**Figure 2:**
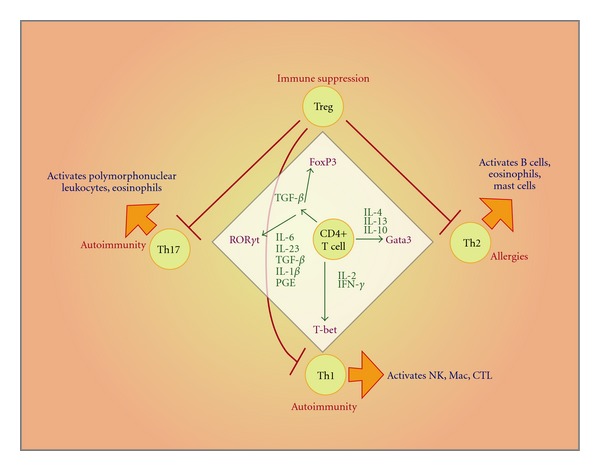
The four different types of CD4+ T cells. The different types of CD4 T cells start from common precursor T cells. Upon stimulation with the different cytokines, the naïve CD4+ cells now get to be selected by various transcription factors. Th1 cells become polarized towards this phenotype in response to IL-2 and IFN-*γ*, and the transcription factor, T-bet, now controls the fate of these cells. In response to IL-4 or IL-13, the Gata3 transcription factor becomes active and Th2 cells result. TGF-*β* now stimulates a common Th17/Treg cell. Upon stimulation with IL-6, IL-23, TGF-*β*, IL-1*β* (in humans), and PGE, Th17 cells become activated through a ROR*γ*T transcription factor. Tregs become polarized by FoxP3. The Tregs have the ability to inhibit Th1, Th2, and Th17 black arrows. The red arrows indicate the effector functions of the various CD4+ subsets. The side effects autoimmunity, allergies, or immune suppression are also noted.

CD4+ T cells can currently be classified into at least 4 different types and each has its own unique function (Figure 2). The different types of CD4 T cells start from common precursor T cells (so-called naïve or ThP cells). Upon stimulation with the different cytokines, the naïve CD4+ cells are selected by various transcription factors in response to various cytokines or other mediators. Th1 cells become polarized towards this phenotype in response to IL-2 and IFN-*γ* and the transcription factor, T-bet, now controls the fate of these cells. These Th1 cells upon activation will release other cytokines that activate cell-mediated effector functions such as CTLs, NK, and macrophages. These effector cells then eliminate cells infected with either intracellular bacteria or viruses. Th1 cells are believed to play major roles in fighting tumors. In response to IL-4, IL-10 or IL-13, the Gata3 transcription factor becomes active and Th2 cells differentiate. Th2 cells activate B cells so that they can make more high-affinity antibodies to help control extracellular bacteria and viruses. The high antibody titers made in response to prophylactic vaccination against these extracellular pathogens are usually attributed to the actions of Th2 cells. Increased IL-4 and IL-5 secretion by Th2 cells stimulates the B cells into producing IgE antibody. IgE in turn causes allergies like hay fever. IgE plays an effector role in fighting large extracellular parasites such as helminths.

TGF-*β* stimulates a common Th17/Treg cell. Upon stimulation with IL-6, IL-23, TGF-*β*, IL-1*β* (in humans), and PGE [93, 94], Th17 cells become activated through a ROR*γ*T transcription factor. Upon activation, Th17 cells play an inflammatory role like Th1 but tend to recruit myeloid cells such as neutrophils, monocytes, and macrophages as their effector cells. These myeloid effector cells control extracellular bacteria, parasites, and fungi. Overactive Th17 cells mediate certain autoimmune conditions. Tregs form as a result of the transcription factor, FoxP3, becoming the dominant transcription factor. The Tregs have the ability to inhibit the actions of Th1, Th2, and Th17 cells. Tregs are essentially brakes that are thought to inhibit an overactive immune response towards any given antigen. Thus, Treg may be a way that produces immunological tolerance towards self.

**(b) Th17 Cells**

Th17 cells have been derived from a lung cancer patient who responded to Mage-A3 [95]. Prostaglandin E_2_ regulates Th17 cell differentiation and if there is an increased amount of PGE_2_ being produced then the presence of these Th17 cells in lung cancer can be explained [96, 97]. In a mouse model that has a metastatic melanoma of the lungs, Th17 cells promote CTL activity towards the cancer and help clear the tumor [98]. The role of Th17 in cancer biology is still not clear since there are reports that Th17 cells help promote cancer development (reviewed in [99]).

Both Th17 and Treg cells possess Aryl hydrocarbon receptors (AhRs) [100]. Activation of this receptor induces transcriptional regulation that controls these cells functions. Depending upon the exact hydrocarbons used, either Th17 or Treg cells can get activated with differing effects. Since tobacco smoke contains many different hydrocarbons, these receptors can easily activate both cell types. AhR activation on lung-derived fibroblasts also induce Cox-2 expression associated with increased PGE_2_ production [101], so it is not surprising that an immunosuppressive environment is being established simply by the constant presence of these tobacco-derived hydrocarbons. AhR is expressed and used by human adenocarcinomas [102] to activate the CYP1B1. This P450 cytochrome enzyme converts noncarcinogens into carcinogens. Presumably, these same enzymes are activated within the precancerous cells that will be eventually turned into tumor cells [103]. AhR can also be found on dendritic cells, and upon activation these dendritic cells have the ability to express indoleamine 2′3′dioxygenase-1 (IDO-1) [104]. Ido-1 is known to inhibit immune responses (see below).

**(c) Tregs**

Th17 and Tregs share a common pathway, in that both require TGF-*β* for early development. IL-6 pushes the development of Th17 cells and IL-23 promotes the growth of already developed Th17 cells. In contrast, Tregs use IL-2 as a growth factor and possess the transcription factor, FoxP3, which drives their maturation and effector function. To eliminate these Treg cells, antibodies towards IL-2R*β* receptor or a recombinant IL-2-diphteria toxin fusion protein (Ontak, denileukin diftitox) [105] can be used to directly kill these cells and enhance immune responses towards tumors. Besides being an alkylating chemotherapeutic drug, cyclophosphamide also kills Treg cells. Tregs inhibit the immune system in part by possessing membrane TGF-*β*. Tregs release soluble TGF-*β* [106]. Treg are thought to be important in downregulating the Th1, Th2, and Th17 cells and perhaps preventing autoimmunity. Thus, it is not surprising that Tregs are found in abundance within lung cancers [107–110]. The increased presence of Treg is thought to explain why many therapeutic tumor vaccines do not work as well as they should in many cancer types.

Ido-1 is an enzyme specifically made by Treg. Ido-1 catabolizes the amino acid tryptophan. Here tryptophan is converted into kynurenine, which limits T cell responses, either because T cells require tryptophan to grow through mid-G1 arrest points or one of its metabolites inhibits T-cell-mediated functions [111–113]. The role of Ido-1 in immune escape mechanisms has been reviewed in Prendergast [114]. Some human lung cancers produce Ido-1, [115]. In *in situ* NSCLC, nine out of eleven cancers were Ido-1+ [113]. In a Lewis lung cancer model, Ido-1 was found to be made by the mononuclear cells infiltrating this tumor or by those cells present in the draining lymph nodes [116]. In human lung cancers, Ido-1 was being made by eosinophils that were infiltrating the NSCLC [117]. Ido-1 expression is strongly stimulated by IFN-*γ* [112]. This finding has significance because CTLs and NK cells could be releasing IFN-*γ*. This would inadvertently stimulate a homeostatic feedback loop that would deactivate the immune system via Ido-1. Ido-1 can be inhibited by using D-1-methyl tryptophan [118], an orally taken drug.

**(d) Myeloid-Derived Suppressor Cells (MDSCs)**

Another type of suppressor cell is called the myeloid-derived suppressor cell. These cells have been recently reviewed by several groups [119–121]. These cells are derived from immature granulocytic or monocytic cells. Some of these cells are stimulated by interelukin-3 (IL-3, [122]), granulocyte-macrophage colony-stimulating factor (GM-CSF), granulocyte colony-stimulating factor (G-CSF), or PGE_2_ [123]. Unlike Treg cells, which can be specifically targeted, MDSCs cannot be controlled as effectively since MDSCs are immature myeloid progenitor cells derived from normal hematopoiesis. Drugs are nevertheless being developed to inhibit MSDCs (reviewed in [124]). In a mouse colon cancer model, use of Cox-2 inhibitors can reduce the negative effects of immature MSDCs [125]. Brandau et al. [126] have shown the increased presence of MDSCs in NSCLCs. These MDSCs release enzymes called arginase [127, 128]. Arginase is a family of enzymes that catalyze the breakdown of arginine into ornithine and urea [129]. Arginase 1 is a cytoplasmic enzyme, whereas arginase 2 is a mitochondrial-derived enzyme. Arginine is needed for proper T-cell function and limits the ability of T cells to respond towards various antigens. Arginine effectively anergizes the T cells thereby making them tolerant towards their environment. Arginine controls immune responses in two opposing ways [130]. Nitric oxide synthase converts arginine into nitric oxide, and this polarizes T cells, DCs, and macrophages into a cell-mediated (Type 1) pathway. PGE_2_ induces macrophages into producing arginase 1, polarizes macrophages into Type 2 cells, and inhibits cell-mediated Th1 immune responses [131]. Additionally, some MDSCs can also produce PGE_2_ [132] as the way they suppress immune responses.

#### 5.3.3. Downregulated Major Histocompatibility Complex (MHC) Makes Lung Cancers Invisible to the T Cells

Lung cancer cells can downmodulate their MHC antigen expression [133–135]. Many lung cancers express very little classic MHC molecules such as HLA-A, -B, or -C. Therefore, CD8 T cells are unable to recognize any tumor antigens that the cancer cells are expressing in the context of MHC class 1 molecules. This also explains why LCs are rarely infiltrated by lymphoid cells [9]. Several pathways have been proposed to explain this loss of MHC expression. Loss of *β*2 microglobulin and loss of transporter of antigen presentation (TAP) molecules [136–138] so that tumor peptides are not loaded successfully onto the MHC are two possible mechanisms to explain this defect. This defect can be corrected by adding cytokines like IFN-*γ* to these cells [139–141]. Thus, once activated Th1 cells can infiltrate the *in situ* tumor and produce IFN-*γ* [141–144]. This released cytokine may fully restore the expression of MHC on the lung cancer cells. However, when good MHC levels are present, beneficial antitumor responses are seen [145].

#### 5.3.4. Increased Expression on Nonclassic MHC: HLA-E, HLA-F, and HLA-G

Tumors frequently express nonconventional MHC alleles such as HLA-E, HLA-F, and HLA-G (reviewed in [146, 147]). The exact role of these nonconventional antigens in tumor biology/immunology of cancers is still unknown. These molecules play a major role in preventing immune rejection of developing fetuses during pregnancy. Cancers may also hijack these molecules as a defense against the immune system. It is thought these nonconventional MHCs produce inhibitory type signals to the CTL or NK and prevent immune responses from occurring. Currently, there are reports that HLA-F and -G are expressed by various lung cancers [148–152]. Soluble HLA-G is reported to prevent proper antigen presenting function [153, 154]. The presence of HLA-E or -G now allows another type of T cells called the *γδ* T cells the opportunity to counter this tumor defense strategy (see Section 7.5).

#### 5.3.5. Counterattack

It is possible that tumor cells can express cell surface molecules, which have the ability to bind to receptors found on immunocytes and induce cell death or anergy. This process is called a counterattack. 

Natural killer (NK) and cytotoxic T cells kill target cells via the release of soluble granzymes and perforin. Perforin essentially pokes holes into the target cells membrane and create an osmotic lysis. The released granzymes can enter these holes and bring enzymatic processes that ultimately result in target cell apoptosis. These cytolytic effector lymphocytes also express a membrane protein called Fas ligand. Fas ligand binds to another cell surface called Fas (also known as APO-l and CD95). CTLs can express Fas ligand (CD95L) and kill cells that are Fas+ [155], which can include lung cancers. Ligation of Fas by antibody induces apoptotic cell death in LC cell lines [156].  Niehans et al. [157] found that 16 of 16 human lung cancers (NSCLCs and SCLCs) expressed Fas ligand. Fas is found on many cell types, including T cells. Ligation of Fas by CTLs, NK or antibodies induces the FADD pathway that leads to caspase 8-dependent apoptosis within Fas+ cells. Two NSCLC cell lines (H2009 and H522) express FasL and have killed the Fas+ Jurkat T cells via a Fas-sensitive mediated cell death pathway [157]. Recently, this concept has been questioned [158]. But Fas ligand also recruits neutrophils into the lung cancers via the production of PGE_2_ [159]. The recruited cells, including MSDCs, may partially explain the overall effect of the counterattack *in situ*. Thus, the exact role of FasL in lung cancer still needs to be fully identified.

One-third of primary lung cancers express a soluble decoy receptor, termed decoy receptor 3 (DcR3) [160]. This decoy binds to FasL and appears to inhibit FasL-mediated apoptosis. The fact that many LCs possess this decoy receptor suggests that Fas/Fas ligand must play an important role in lung cancer defense.

## 6. Impediments towards Lung ****Cancer Immunotherapy

Many arguments can be made against treating lung cancer with immunotherapy. Nonsmokers see this as a cancer that the smokers gave themselves due to their bad habit. Nonsmokers may consequently argue that developing immunotherapy for smoking-related lung cancers is a waste of time when there are many other types of nonsmoking-related cancers to treat. Hence, research funding is harder to procure for lung cancer. On a biological level, LCs have a variety of defense mechanisms: soluble mediators: transforming growth factor-*β* and cyclo-oxygenase-2, which makes prostaglandin E, interleukin-10, and arginase; defensive molecules such as Fas ligand, program death ligand-1 (B7-H1), nonconvention HLA molecules, lack of major histocompatibility (MHC) class I molecules, and recruitment of suppressor type cells. All these obstacles can naturally limit immune responses towards these cancer cells. These same arguments were also made against the immunotherapy of malignant human gliomas [161]. But progress is now being made against gliomas using dendritic cells pulsed with the patients' autologous cancers and other immunotherapies [1, 162, 163]. Thus, the development of immunotherapy towards lung cancer is significantly behind that observed with other types of immunotherapy for cancer.

## 7. Immunotherapy against Lung Cancers ****and Opportunities for Intervention

### 7.1. Early Steps

Since the beginnings of modern medicine, doctors have been looking for the “magic bullet” to treat tumors, whether it is through drugs, surgery, radiation, or other modalities. Back in the late nineteenth century William Coley discovered that a number of cancer patients who simultaneously had bacterial infections sometimes had miraculous cures to their cancers. He later used the bacteria isolated from those miraculous cures as toxins and his treatment was known as “Coley's toxins.” Many explanations are possible, that is, released cytokines (IFN-*γ*, TNF), CpG segments of the bacterial DNA, and bacterial cell wall products, like LPS, can activate the toll-like receptors (TLRs) found on immunocytes, and so forth. Thus, biological response modifiers can enhance oncological therapies. Bacillus Calmette Guerin (BCG) was initially proved to be effective at treating superficial bladder cancer [164]. Injecting these bacteria into bladder cancers is now a routine therapy. This induced inflammation not only kills the tumor, probably by activating the innate immune system, but also leads to sustained immune responses by activating the local antigen-presenting cells. BCG was tried to treat lung cancer but failed to show any responses [165–167]. Two other therapies such as using *Corynebacterium parvum* [168] and *Mycobacterium vaccae* [169] have been tried with lung cancers. Both of these therapies initially seemed to fail. However, when the data concerning the Mycobacteria vaccae was reevaluated, there was a higher survivor rate in patients who successfully completed the therapy than in those who did not [170]. Since compliance was a major issue with this therapy, it probably mean that many toxicities were occurring. The consequences of these toxicities probably means that it cannot be developed any further.  Table 2 summarizes the various types of lung cancer immunotherapy that are currently being used to treat lung cancers.

Hollinshead et al. [171] showed significant 5-year survival rates for all types of lung cancer patients treated with a tumor antigen subunit vaccine purified from different types of lung cancers. The cancer vaccine was made by isolating the tumor antigens by an affinity chromatography technique using a variety of antibodies directed towards the antigens found on lung cancers. The 5-year survival of 234 treated patients with stage I and II patients was 69%, compared with 49% of the untreated controls. Unfortunately, this work did not proceed, despite its early promising success, since this vaccine was deemed dangerous. This vaccine was made with Freund's Complete Adjuvant (FCA), which contains mycobacterial proteins. These bacterial proteins stimulate the innate immune system via toll-like receptors (TLRs). The protocol included 3 vaccinations a month apart with FCA. FCA causes severe immune reactions and has painful reactions. Tissue damage such as ulceration is one potential sequela. These toxicities probably limited the further use of this promising protocol. This work did indicate that active immunotherapy can successfully treat some LC patients.

### 7.2. Direct Antibody Therapies

In the mid-1970s, Kohler and Milstein created hybridoma technology, which led to the creation of monoclonal antibodies. In the early to mid 1980s, this technology was commercialized for generating clinically applicable antibodies. Monoclonal antibodies were used to directly treat cancers early on. These antibodies were thought to be the “magic bullets” that could kill tumor cells. The advantage was that these antibodies could be easily scaled up, because they were derived from immortalized hybridoma cell lines. These antibodies could be used alone as unmodified antibodies to allow antibody-dependent cytotoxicity (ADCC) to occur. These antibodies could also be conjugated with radioisotope, chemotherapeutic drugs, or with cytokines or enzymes to target the cancer. Monoclonal antibodies are very unique to the tumor and it was thought they should only home towards the tumor and thereby limit toxicity to other cells. However, in retrospect, antibodies do not penetrate deeply within the tumor bed since they bind more heavily to the peripheral tumor cells that are initially exposed to the antibody.

The CC49 monoclonal antibody, which binds to a tumor-associated glycoprotein 72 (TAG-72), has also been tried in conjunction with radioisotopes [172]. Antibodies directed towards a cell surface ganglioside, called GD3, are being tried. GD3 is highly expressed in SCLC, but not in NSCLC. Bec2 is an anti-idiotypic antibody that binds to the idiotype of the antibody against GD3 and so it is thought to be a mimic of GD3. This antibody was combined with a BCG adjuvant to treat SCLC. These clinical studies against SCLC proved to be somewhat positive when compared to historic controls [173]. A similar approach was taken towards a Neu-glycosylated sialic acid ganglioside, called NeuGc-GM3, that is found on all types of LC. The anti-idiotypic antibody was called 1E10. This antibody was tried in both SCLC and NSCLC [174]. A survival benefit of about 6 months was noted in those patients that developed immunity against NeuGc-GM3. 

The first antibody (early 1990s) that seemed to have any major clinical effect against any type of cancer was the herceptin (Trastuzumab) antibody, which targets the her2/neu surface protein that was heavily overexpressed on some breast cancers [175]. At the time of its initial discovery, it was thought that this antibody would simply bind to a surface protein found on the cancer cell. Afterwards, it became apparent that the success was due to the fact that this antibody was interfering with a key cell-signaling pathway that prevented a growth factor signal pathway from being activated on the her2+ cells. The fact that this antibody also allowed antibody-dependent cell cytotoxicity (ADCC) probably helped its therapeutic efficacy [176]. So the take-home lesson is that it is vital to target a key biological factor that controls a unique aspect of the tumor and not just target any random cell surface tumor protein. Thus far, no antibody that directly targets an equivalent function of her2 on LC has been found. However, the creativity of medical scientists and the versatility of antibodies have allowed certain antibodies to be developed that enhance results to cancer to be developed and used in an indirect method (see Section 8).

### 7.3. Lymphokine-Activated Killer (LAK) Cells

Later immunotherapy studies focused on using a variety of other more advanced *ex vivo* cellular techniques, such as using lymphokine-activated killer (LAK) cells, cytotoxic T lymphocytes (CTLs) derived from tumor infiltrating lymphocytes (TIL) cells or from draining lymph nodes. The hope here was to generate overwhelming numbers of effector lymphocytes *in vitro* that could be applied *in vivo*. The genetic revolution of the 1980s and 1990s provided cancer immunologists with the opportunities to acquire sufficient amounts of cytokines and growth factors to stimulate the immune system on a large clinical scale. Some of the early cytokines that were genetically cloned and tested therapeutically were interleukin-2 (IL-2) and tumor necrosis factor (TNF). LAK cells became very popular in the mid-1980s. Human peripheral blood lymphocytes upon stimulation with IL-2 (a lymphokine known back then) turn NK and CD8+ T cells into nonspecific killer cells that lyse tumor cells *in vitro *[177]. When the cells were infused back into the patient, a clinical response was sometimes observed against melanomas and renal cancers [178]. One severe limitation of this therapy was that systemic toxicities occurred in many patients and this prevented many recipients from completing their therapies. This was perhaps due to the cytokines the LAK cells released such as TNF or IFN-*γ*. Enthusiasm for this modality also ran out when it became apparent that clinical responses were equally found when IL-2 was just administered *in vivo* alone without having to go through the laborious LAK cell collection and processing protocols [179]. Even though lung cancer cells were nonspecifically killed *in vitro*, LAK therapies with lung cancer patients largely failed. The review by Al-Moundhri and colleagues [180] covers the results of LAK therapy with lung cancers that was acquired from the 1980s.

Attempts were also made where advanced NSCLC patients were directly injected with IL-2 and TNF intratumorally [181]. This was a method of stimulating endogenous LAK cell precursors already within the tumor. Most patients experienced severe toxicities. Three patients had partial or stable disease that only lasted 6–9 months, but 1 patient with severe metastatic disease did live for at least 30 months [182].

### 7.4. Tumor Infiltrating Lymphocytes (TILs) and Draining Lymph Node (DLN) T Cells. 

The next great hope for immunotherapy involved isolating and expanding the tumor infiltrating lymphocytes (TILs). One early murine study fueled support for this concept [183]. This approach was novel because both CD4+ and CD8+ T cells could be isolated and expanded. The theory was that these lymphocytes should be specific for the tumor since they were already present within a tumor. This occurred in the days before the importance of antigen presentation by dendritic cells was known (see Section 7.6). It was observed that T cells needed to be “antigen-dosed” every 2 weeks or so, otherwise these T lymphocytes lost antigen specificity. There is a paucity of lymphocytes within many lung cancers [9]. So a derivative of this TIL approach was to take the lymphocytes from the tumor draining lymph nodes (DLNs) and expanding those cells *ex vivo* as you would do with the TIL. Both TIL and DLN cells were viewed to be very tumor specific and would have less toxicity than the LAK cells. Occasionally, these types of cells could have antitumor immune responses against cancers including lung [184, 185], but in most cases these cells did not work. With the subsequent discovery of Treg cells, the presence of these Tregs now explains why many of these TILs and DLNs expanded cells did not work as well as they were hoped because they were selectively enriched for Treg using the IL-2 (see Section 5.3.2(a)).

However, a big advance came about when it was discovered that if nonmyeloablative treatment was given to cancer patients before they received TIL cells, better clinical responses were seen [186]. Here other lymphocytes were killed, including Treg and other non-tumor-specific T cells. This mass killing of these lymphocytes then provided niches for these reinfused *ex vivo* expanded cells [187]. 

### 7.5. *γδ* T Cells

Another T cell type also matures in the thymus, besides the classic *αβ* T-cell receptor (TCR) rearranged T cells. These cells instead use a rearranged *γδ* TCR to recognize their antigens. These *γδ* T-cell receptors have a very restricted TCR diversity but are not MHC restricted. These cells may recognize nonclassic HLA-E and HLA-G molecules through NKG2D or V*γ*9V*δ*2 receptors. These lymphocytes were initially discovered to be cytotoxic towards leukemia cells. Wrobel and colleagues [188] discovered that these cells also had the ability to recognize and kill NCSLC lung cancer cells *in vitro*. Several of the ligands that *γδ* T cells can recognize are MICA, MICB, ULBP-2 and ULBP-3 binding proteins found on the lung cancers [188]. Groh and colleagues [189] showed that *in situ* lung cancers possessed some ligands that *γδ* T cells recognized; these *γδ* cells were found *in situ* with the lung cancers. This non-MHC-restricted killing by *γδ* T cells opens up the possibility that allogeneic donors could be used for therapeutic purposes in lung cancers without risking the possibility of graft versus host reactions or autoimmune diseases. Clinical trials using *γδ* T cells against recurrent NSCLCs are beginning to appear [190]. Ten patients were expanded with their autologous *γδ* T cells, the median survival of these treated NSCLC patients was 401 days. Thus, this adoptive form of immunotherapy was deemed safe.

### 7.6. Gene Therapy

Gene therapy became the next big topic for a decade starting in the early/mid-1990s. The seminal work of Dranoff and colleagues [191] showed that immune-mediated rejection was not the same as generating long-lasting immunity. When living B16 melanoma cells were transduced with IL-2 or TNF and injected into mice, no tumor growth occurred as a result of CTL and NK becoming activated by those released cytokines. In contrast, IL-4- or GM-CSF-transduced cancer cells formed tumors when injected subcutaneously. However, if the IL-4 or GM-CSF transduced cells were irradiated and then used as a prophylactic vaccine, long-term memory was generated against the unmodified B16 tumor cells. Mice that rejected the IL-2- or TNF-transduced B16 melanoma cells showed no lasting recall memory, even though an immune response rejected the initial IL-2- or TNF-transduced tumor cells. Later it was realized that the released IL-4 and GM-CSF stimulated/recruited a poorly understood cell, which at the time was called the dendritic cell (DC). Today dendritic cells are considered the best antigen-presenting cells in the body. DCs can stimulate both naïve and previously activated T cells. This genetic engineering work produced a major paradigm shift that revolutionized our concepts in cancer immunology and has opened up the possibility of using immunotherapy against many different types of cancers.

Lung cancer vaccines transduced with various cytokines and costimulatory molecules have been used clinically. GM-CSF-transfected lung cancer cells used as vaccines are the most commonly used ones. Salgia et al. [192] was the first to use an autologous NSCLC tumor cells were transfected with an adenovirus that delivered GM-CSF. They used this in 97% of their patients. Here the tumor cells were isolated and transfected with adenoviruses. Two of the treated patients were noted to have been disease-free for 42+ months. A larger study was later run and was known as a GVAX approach [193]. The transduced cancer cells are irradiated and then used as a whole-cell vaccine. The longest surviving patients were noted to have received the most cells, which also had the highest expression of GM-CSF. A follow-up trial, called the allogeneic GVAX approach (allo-GVAX or Bystander GVAX) [194], was performed where allogeneic K-562 cells, which secreted much more GM-CSF than the autologous lung cancer cells, were combined with unmodified lung cancer cells. The results proved to be negative in terms of patient responses towards the cancers. One possibility is that this elevated dose of GM-CSF induced MDSC, which hindered antitumor immunity (see Section 5.3.2(d)). In conclusion, using autologous lung cancer cells that were transfected with the GM-CSF was the most beneficial vaccine.

Another genetic approach was to engineer lung cancer tumor cells with either HLA-A1 or HLA-A2 MHC molecules along with the immune costimulatory molecule, CD80. The idea here was to use a whole cell line (AD100) that expressed more MHC class 1 with the costimulatory molecules [195] to stimulate endogenous T cells directly by the vaccinated cells. Of their 18 patients tested, the median survival time was 18 months. No differences were noted in the responses of their patients to HLA compatibility, so this finding suggests that cross-presentation of tumor antigens was occurring, so HLA matching of the vaccinating tumor with the patient was not necessary to generate clinical responses.

A different genetic approach was taken with the canary pox virus. Here the virus genome was modified so that it would deliver a lung cancer antigen called the carcinoembryonic antigen (CEA) along with the B7.1 costimulatory molecule. This construct was called the ALVAC [196]. CEA is overexpressed in roughly 70% of NSCLC. This vaccine was injected intramuscularly every 4 weeks for 3 months into lung adenocarcinoma patients. No toxicities were seen with the highest doses of the virus given. Three patients had stable disease that correlated with CEA-specific T cells that produced IFN-*γ*. This project did not proceed any further with lung cancer but seems to be proceeding further with colon cancer.

CEA is used as a tumor vaccine with the common yeast, *Saccharomyces*, being used as the delivery vehicle. GlobeImmune (Louisville, Colorado) has pioneered this “Tarmogen” approach. Their clinical product is called GI-6207 and is used with metastatic adenocarcinomas. A phase 1 study enrolled 25 patients with three doses, which was administered at 4 sites subcutaneously biweekly for three months then monthly until disease progression. Twenty percent of the patients had stable disease and had declines in serum CEA levels [197].

The Mucin-1 (Muc-1) antigen is a core peptide of a glycoprotein found on many epithelial cancerous cells, including NSCLC. Muc-1 is thought to play several roles in cancer including loss of immune recognition, tumor cell migration, and resistance to apoptosis [198]. The attenuated Ankara strain of vaccinia virus was genetically engineered to transduce the Muc-1 antigen along with the IL-2 gene to create the “TG4010” vaccine. The vaccine was administered weekly by subcutaneous injections at the dose of 1.0 × 10^8^ PFU and then once every 3 weeks until disease progression. There was an improved clinical outcome with TG4010 in patients, especially in those having T lymphocytes displaying an activated NK phenotype [199, 200]. The higher levels of activated T lymphocytes also correlated with longer TG4010 patient survival than the chemotherapy alone controls. In addition, increased circulating IFN-*γ* levels predicted a longer survival for the TG4010-treated patients.

### 7.7. Peptide Vaccine

A number of tumor-associated antigens have been discovered in lung cancers. Van der Bruggen et al. [201] have compiled a listing of various tumor antigens that have been found within human lung cancers. These tumor-associated antigens are composed of mutations, shared tumor-specific, differentiation, and overexpressed antigens. These antigens could be possible antigens used for lung cancer immunotherapy. 

A synthetic 25-amino-acid Muc-1 peptide was formulated into a liposome and is called L-BLP25 or Stimuvax [202]. This immunogen is now being used as a vaccine in NSCLC. The vaccine was injected into patients that received a single dose of cyclophosphamide. Sixteen of 65 patients demonstrated a T-cell immune response, and the patients had median survival time of 30.6 months compared to 13.3 months with the best supportive care.

The Wilms tumor antigen-1 (WT-1) is found within most NSCLCs and SCLCs [203]. Oka and colleagues [204] used a 9-mer of WT-1 (which is restricted for the HLA-A2402 allele) and emulsified it with the montanide ISA51 adjuvant. They administered this vaccine three times at 2-week intervals to breast, leukemia, and lung cancer patients who were HLA-A2402 positive and had WT-1-positive tumors. Three of the 10 lung cancer patients showed an immunological response as defined by a positive tetramer staining profile along with elevated intracellular IFN-*γ* expression. One patient has managed to survive the lung cancer and has been repeatedly vaccinated during this time (>2+ years).

Cyclophilin B was found on lung cancer adenocarcinomas and can be a target of CTLs [205]. Gohara and colleagues [206] used a cyclophilin-based peptide vaccine. Peptides were mixed with incomplete Fruends adjuvant (IFA) and injected as a subcutaneous vaccine in a phase I study in Japan. No significant increases in cellular responses were seen and this study was deemed to have failed.

The Mage-A3 peptide coupled with the AS02b adjuvant was tried in 182 patients that were Mage-A3+ NSCLC [207, 208]. This trial was using the GlaxoSmithKline MAGE-A3 protein. Some trends suggested beneficial results occurred and prompted further studies. These positive results initiated the development of the GSK1572932A study (ClinicalTrials.gov) and is part of the MAGRIT (MAGE-A3 Adjuvant Non-Small-Cell Lung Cancer Immunotherapy) study [209]. This study was opened in 2007 and is now closed to enrollment. This vaccine was composed of 13 intramuscular injections of the vaccine. Survival statistics are currently being collected to determine if these results are truly significant.

The IDM-2101 composite vaccine is made by IDM Pharma (Irvine, CA). This synthetic peptide vaccine is based upon 10 different HLA-A2-restricted epitopes against 5 different antigens (CEA, p53, Her2, Mage-2, and Mage-3 antigens along with a pan-DR epitope). A phase II study was done [210]. Survival was longer (17.3 months) in patients demonstrating an immune response to epitope peptides (*P* <  .001) than those not immunologically responding. One patient had a complete response to the vaccine.

Epidermal growth factors (EGFs) are frequently overexpressed in LC and their receptors are frequently mutated within LC [211]. Hence, small chemical inhibitor strategies are targeted to the EGFR pathways and are frequently used in LC [212]. In this vaccination strategy, a recombinant fusion protein of EGF was conjugated to a bacterial P64K protein as a carrier protein to induce immune responses. As a result of this vaccination there was an increased titer of circulating anti-EGF antibody titers. This also correlated with a decreased level of serum EGF. They also made a direct correlation between antibody responses with patient survival, especially in those patients younger than 60 years old. This age response is very important, since as people age their immune responsiveness decreases. So vaccination may not be effective for individuals older than this age [213]. The data from 3 studies was complied in a meta-analysis and confirmed the study above [214].

### 7.8. Dendritic Cell Vaccines 

Most smoking-related cancers have p53 mutations. DC-based vaccinations were based on infecting DC with p53 transfecting adenoviruses [215]. *In vitro*, when these transfected DCs are activated they can generate CTLs versus p53 [216]. These trials showed some progress. Introgen Therapeutics (Austin, Tx) in collaboration with the previous group is developing this concept with the INGN225 vaccine just with the p53 gene. In SCLC, this therapy induced a significant immune response and sensitized the SCLC to subsequent chemotherapy [217].

Hirschowitz and coworkers have developed an allogeneic 1650 adenocarcinoma cell line that was characterized for expression of Her2/neu, CEA, WT-1, Mage2, and survivin. They used these apoptotic cells to load immature autologous CD14+ monocytic DC stimulated with GM-CSF/IL-4. These DCs (80–90 million DCs) were injected as an intradermal vaccine to stimulate the immune system in a variety of stage IA to stage IIIB NSCLC patients [218]. Antigen-specific immune responses were noted in this study in the majority of the 16 patients tested. A follow-up study of these patients along with 14 new patients was reported 3 years later [219]. Many of these patients were still alive, although it was not clear whether these positive responses were due to good surgical resections or due to immunotherapy.

Dendritic cells are beginning to be developed as a therapy in China, which are pulsed with the Xage-1b protein [220]. Xage-1b is a member of the cancer-testis family of antigens and is overexpressed in many lung cancers. In early studies, this methodology does generate CTLs *in vitro *and has the ability to kill lung cancers, but not normal lung cells. Thus, this antigen might be added to the tumor antigen armaments towards lung cancer.

Tumor lysates derived from autologous NSCLCs are being electroporated into dendritic cells in Korea. These DCs were then injected into advanced NSCLC patients. In these early studies, Um and coworkers [221] showed that when their patients received the most dendritic cells (12 million cells) 3 times at 2-week intervals, five out of the nine patients resulted in increased IFN-*γ* production after an *in vitro* restimulation. Two of the patients treated with these cells appeared to have some beneficial effects. So getting tumor antigens into dendritic cells can be effectively done in a couple of ways.

When people view tissue culture cells under the microscope, one can frequently see remnants of cells, left as a cell moves away. These released cell-debris particles are called exosomes. These exosomes contain all the small material as the cell that produced them, proteins, RNA, microRNA, and so forth, including tumor antigens. Some of these exosomes when formulated with CpG adjuvant and injected into animals can create immune responses towards the original tumor [222]. This intriguing observation was followed up when exosomes from mature dendritic cells were used as the vaccine [223–225]. This work has now been developed into a clinical modality for treating NSCLC at the Institut Gustave Roussy in France. Drs. Besse and Chaput are spearheading this approach. In this trial autologous DCs are being loaded with HLA-DP04-restricted MAGE-3, and HLA-A02-restricted peptides NY-ESO-1, MAGE-1, MAGE-3 and MART-1. So far, no results of this clinical effort have been reported.

### 7.9. Knockout Strategies

The advantage of using a whole cancer cell is that the entire spectrum of tumor antigens can be harnessed against the tumor; all these antigens can now stimulate multiple clones of T cells. This contrasts with vaccination strategies that only use a few antigens, such as the peptide vaccines or adenoviruses transfecting tumor antigens. For immune responses to occur, APC must take up the antigen and chop it up via the DC proteosome. These digested peptides should then be presented via the patient's own DC and stimulate the host T cells. In theory, any tumor cell could be used to vaccinate the patient, regardless of the patient's HLA profile, as the Raez et al. study [195] seems to show. NovaRx (San Diego, CA) has genetically knocked down TGF-*β*_2_ expression and is using a combination of 4 allogeneic cell lines (lucanix also known as belagenpumatucel-L) as a vaccine [226]. Since TGF-*β* is prevented from being made, when these killed tumor cells are taken up by the DCs they should respond maximally, since no endogenous TGF-*β* was present to hinder DC function. Hence, better immune stimulation should occur. Early results in a phase II study do suggest the vaccine is well tolerated. It also generated T cell reactivity in 11 of 13 of these treated lung cancer patients. As a result, survival of these immune responding patients was 32.5 months compared to 11.6 months with the nonresponders. Thus, allogeneic cells do seem to show they can act as an effective immunogen, although it is still really too early to make it an established fact.

## 8. Antibodies That Can Augment ****Immune Responses

In contrast to antibodies being used to directly treat the cancer, antibodies can create positive clinical responses in other ways. One strategy of antibodies is to either use them as an antiangiogenic approach or use antibodies (anti-CTLA-4, anti-PD-1/PD-L1, and anti-IL-2 receptors) to inhibit some of the negative regulatory pathways. The former antibody therapy is beginning to show clinical responses in many cancers, including NSCLC, while the later antibodies may be added to other therapies as discussed earlier. These combined therapies could have a potentially big impact on lung cancer immunotherapy. So far, none of these approaches has been reported with lung cancer. But we anticipate we will see more of these types of studies in the near future.

### 8.1. Bevacizumab Is an Anti-VEGF Antibody Developed by Genentech. 

VEGF, as described in Section 5.3.1(c), cannot only play a role in tumor angiogenesis by working on the tumor recruitment of endothelial cells but may also play a role in breaking autocrine loops in LC. VEGF can inhibit immune responses by turning off the actions of dendritic cells. Bevacizumab has recently been approved by the US FDA as a first-line therapy for metastatic colorectal cancer. And it also does appear to be effective against other human cancers, too [227]. In a phase 3 study done with metastatic NSCLC patients, there was increased progression-free survival (*P* < 0.0001). Additionally, there was a better overall survival (12.5 months compared to 10.2 months) with a *P* < 0.007. So some progress is now being made against lung cancer using this antibody. 

### 8.2. Ipilimumab Is the Antibody That Targets an Immunomodulatory Molecule Called CTLA-4. 

As T cells become activated into effector cells, they are induced to express CTLA-4 antigen, which is capable of binding to the costimulatory molecules found on DC, CD80, and CD86. CTLA-4 binds better to the CD80/CD86 molecules than does the immunostimulatory CD28 receptor found on the T cells [118]. Negative signals are delivered to the T cells upon binding to APC CD80/CD86 molecules via CTLA-4 and the T cell is essentially inhibited from further functional activities. These T cells will eventually be eliminated via apoptosis. Both natural and induced Tregs also use CTLA-4 (cytotoxic T lymphocyte antigen-4) to inhibit immune responses by inhibiting APC function. Antibodies towards CTLA-4 such as ipilimumab are being developed to inhibit this pathway to improve tumor vaccines in humans. This antibody is now being used in NSCLC [228]. By preventing this CTLA-4-mediated downregulation, an enhanced immune response can be made and can probably enhance antitumor immune responses. Recently, this antibody has been successfully used for the treatment of melanoma [229]. Here, additional, four months of survival were noted in these patients. In general, these antibodies have to be carefully watched since they have the potential to cause autoimmunity and produce other severe effects that limit their therapeutic ability.

### 8.3. Another Family of Antibodies Is Directed towards the Family of PD-1/PD-L1 Inhibitory Molecules [230]

The PD-1/PD-L1 system can also be considered a tumor counterattack strategy. The programmed death-1 (PD-1) molecule is a member of the CD28 family. The ligands for PD-1 (PD-Ls) are PD-L1 (B7-H1) and PD-L2 (B7-DC). Upon signal transduction with these proteins the T cells become triggered for cell death via apoptosis. PD-L1 has been detected on human lung cancers. Dong et al. [231] showed 95% of the lung cancers (20 of 21, including adenocarcinomas, squamous and small cell) were positive for B7-H1(PD-L1). Iwai and colleagues [232] also showed a similar type of mechanism. Brown et al. [233] also showed 6 of 6 adenocarcinomas and 8 of 8 squamous cancers were strongly positive for PD-L1(B7-H1). When *in situ* lung cancers expressed more B7-H1, there were fewer T cells present than in the B7-H1 negative cancers [234]. Perrot et al. [235] also showed that the myeloid dendritic cells that infiltrated NSCLC were blocked in an immature state and were B7-H1+. 

Besides the B7-H1 and B7-DC molecules, a couple of other family members with similar biological functions, namely B7-H3 and B7-H4, are expressed on various NSCL cell lines. These markers are found within *in situ* squamous and large cell carcinomas [236, 237]. Roughly half of lung cancers expressed at least one of these markers.

A variety of antibodies from various companies, Bristol-Myers Squibb, Merck, GlaxoSmithKline, and Cure Tech, are targeting these receptors/ligands, which also can inhibit T cell and NK cell responses. One interesting fact is that these antibodies do not have as much toxicity as the anti-CTLA-4 antibodies seem to possess [124]. Thus, this antibody will probably be the best way to target the immunosupressor cells. 

### 8.4. The Previous Use of an ONTAK Immunotoxin Which Depletes Treg [238] via the High-Affinity IL-2 Receptor (See Section 5.3.2(a)) Is Another Way to Target This Same Receptor

*Daclizumab* [239] targets the high-affinity interleukin-2 receptor-*α* on Treg cells. By eliminating Treg cells, a more sustained antitumor immune response can also be maintained. This therapy is currently being tried in melanoma patients at the University of Chicago and in glioma patients at Duke University. Here the idea is to eliminate the Treg before they inhibit the optimal anti-cancer immune response. Since LC has high concentrations of Treg, this could prove to be very good at improving clinical results.

## 9. Summary

Education is often considered a painful process, and that is certainly true in the case of lung cancer. Each new lesson requires expensive clinical trials to learn this vital information. Each cancer has its own unique set of tricks to avoid clinical therapies. Lung cancers use a variety of defensive strategies to escape chemo- and radiation therapies also can serve double duty by resisting the immune system. Immunologists are beginning to design rational therapies to counteract these defensive strategies. It will still take work to develop effective therapies against this killer cancer. Most likely, no single immunotherapy will work as a stand-alone therapy and it will have to be combined with other therapies to achieve a cure. Progress with immunotherapy is slowly being made against other cancers previously considered terminal cancers, that is, melanoma, glioma, and castrate-resistant prostate cancer. The lessons learned in those clinical trials can certainly be applied towards lung cancer.

## Figures and Tables

**Table 1:** Mechanisms of lung tumor escape.

(1) Antiapoptosis genes	
(a) Bcl-2, survivin	
(b) Loss of apoptosis effector molecules: caspases, p53 family	
(2) Drug resistance genes	
(a) Multidrug resistance proteins	
(b) CD133	
(3) Immunoresistance genes	
(a) Soluble factors: PGE, VEGF, TGF-*β*, Ido-1, arginase	
(b) Immunosuppressive cells: Treg and MDSC	
(c) Loss of classical MHC and/or gain of nonconventional MHC	
(d) Counterattack: Fas ligand and PD-L/B7-H family members	
(e) Age and loss of functional immune system	

**Table 2:** Types of immunotherapy for lung cancer.

(1) Active vaccination: subunit vaccines from tumor lysates	
(2) Passive antibody administration	
(a) Direct tumor binding: various tumor antigens	
(b) Indirect approaches: anti-VEGF, anti-CTLA-4 anti-PD-1/PD-L1, and anti-Treg	
(3) Passive cell-mediated administration	
(a) LAK cells	
(b) TIL/draining lymph nodes T cells	
(c) *γδ* T cells	
(4) Gene therapy	
(a) IL-4	
(b) GM-CSF	
(c) MHC and/or costimulator molecules	
(d) TGF-*β* knockdown	
(5) Dendritic cell vaccination	
(a) Peptides	
(b) Tumor lysates	
